# The body mass-maximum speed relationship and the athletic capability of giant proboscideans and sauropods

**DOI:** 10.1038/s41598-025-32536-3

**Published:** 2025-12-24

**Authors:** Javier Ruiz, Anthony Romilio, Juha Saarinen, Angélica Torices, Juan Manuel Jiménez-Arenas

**Affiliations:** 1 Departamento de Geodinámica, Estratigrafía y Paleontología, Universidad Complutense de Madrid, Madrid, 28040 Spain; 2 School of the Environment, The University of Queensland, Brisbane, QLD Australia; 3 School of Veterinary Science, The University of Queensland, Gatton, QLD Australia; 4 Department of Geosciences and Geography, University of Helsinki, Helsinki, Finland; 5 Departamento de Prehistoria y Arqueología, Universidad de Granada, Granada, 18071 Spain

**Keywords:** Palaeontology, Biomechanics

## Abstract

Estimating the maximum speed capability (athletic capability) of very large fossil animals is challenging. While large animals have limbs favoring longer stride lengths and higher speeds, their body mass imposes limitations on bones, joints, available forces, and physiology, resulting in the fastest animals not being the largest. Here we use the well-known relationship between body mass and potential maximum speed to calculate upper limits for the athletic capability of fossil giant proboscideans and sauropods. First, we assess the reliability of two different fits to maximum speed data for mammals. Subsequently, we analyze masses and speeds of live proboscideans, finding they consistently move below the lower bounds predicted by model fits. Finally, we propose maximum speed for representative fossil proboscideans and sauropods. Although calculated maximum speeds for larger proboscideans are comparatively lower, there is substantial overlap with those observed in live proboscideans. For the largest sauropods approaching or exceeding 50 Tn our calculated maximum speeds are around 10 km/h or lower. These findings suggest that immense body size and graviportal structure of sauropods were key factors likely restricting their locomotion to a single, steady gait, consistent with fossil trackway evidence.

**Supplementary Information:**

The online version contains supplementary material available at 10.1038/s41598-025-32536-3.

## Introduction

Estimating the athletic capability (i.e., maximum speed; see ref^1^.) of fossil animals is, in the absence of direct observations, a difficult task, moreover when dealing with very large (one ton or more) ones. Calculating speed from fossil footprint trackways is a widely used methodology^2–5^, but it is usually applied to slow-moving animals (representing typical displacement behavior), and clear evidence of running gaits comes from comparatively light animals^6,7^, which are likely moving below their maximum potential speeds. Numerous works have estimated bone parameters or properties in order to determine safety conditions and upper limits for the speed that large dinosaurs could have reached, finding in general modest results^8^.

Speed estimates based on fossil trackways of large extinct trackmakers are often used to infer their locomotor capabilities, however, these calculations generally reflect the slow-movement behaviors typical of large-bodied animals^9–11^. For very large sauropod and theropod dinosaurs^1,12,13^ various factors limit their potential speed. Their massive size imposes substantial stress on bones and joints, while the scaling of muscle power relative to body mass results in disproportionately high energy costs and structural strain during rapid movement^1,14^. Consequently, large dinosaurs likely prioritized slower-paced movement, with biomechanical constraints limiting their capacity for high-speed locomotion.

There is a well-known relationship between body mass and potential maximum speed in animals^1,15–20^. Whereas large animals have large limbs that potentially favor longer stride lengths and higher speeds, large body mass also imposes limitations through several ways, such as overburden on bones and joints, physiological costs or inertial forces^1,20–22^; thus, extant fastest animals are not the largest (e.g., cheetah^23^ and pronghorn^24^).

Garland^15^ first proposed an empirical regression between maximum speed and body mass for land mammals. Subsequent works by Dick and Clemente^19^ and Hutchinson^1^ presented improved regressions from increased and modified databases. These works demonstrated that there is a maximum speed which would occur for body masses below 100 kg. Hirt et al.^20^ developed a model for maximum speeds considering time-dependent acceleration from muscle force and energy availability. This model found that maximum speeds for terrestrial locomotion occurred at body mass slightly below 100 kg. In this work we analyze the application of both the Garland-Dick and Clemente-Hutchinson regression (hereafter the G-regression) and the Hirt et al.’s model (hereafter the H-model) to calculate maximum speeds for large land animals, specifically for giant proboscideans and sauropod dinosaurs.

First, we assess the deviation of animal terrestrial vertebrate maximum speeds data from the predictions from both body mass-based procedures. Then, we use a database of masses and speeds of live proboscideans for guide maximum speed calculations for these animals. Finally, we use our results to propose maximum speed values for large graviportal fossil animals (animals with short limb bones and robust skeleton^1^, adapted for weight-bearing, with column-like limbs supporting massive body weights) as a function of their body mass, and compare and discuss our results with those of previous studies.

## Results

### Body mass-maximum speed relationships

Here we use the version of Hutchinson^1^ of the database of maximum speed and body mass for land mammals (this author amended maximum speeds for several large animals), which included for 150 mammals, with body masses ranging from around 9 g to 6 tons. We further amended the database (see Supplementary Table 1) to adjust the data for including the fastest Asian and African elephants from a data base obtained in controlled conditions^25^, for consistency with the data for live elephants used for guide our maximum speed calculations for proboscideans. Thus, a polynomial second-order fit with logarithmical terms can be obtained (which is very similar to that presented by Hutchinson^1^). Expressing the body mass in tons (because we are dealing in this work with very large animals) and the speed in km/h (as usually used in vertebrate paleontology), the modified regression fit is

1 $$Spee{d_G}({\mathrm{km/h}})={\mathrm{antilo}}{{\mathrm{g}}_{{\mathrm{10}}}}\left[ {1.6347 - 0.1517({\mathrm{lo}}{{\mathrm{g}}_{{\mathrm{10}}}}M) - 0.0615{{({{\log }_{10}}M)}^2}} \right],{\text{ }}[{R^2}\,=\,0.76]$$

where antilog_10_(*x*) = 10^*x*^ and *M* is the body mass. This regression gives a maximum speed around 65 kg. In order to evaluate the predictions from Eq. (1) we obtain the mean and standard deviation of the ratio between the observed and predicted speed for all 150 mammals in the database, obtaining (see Supplementary Table 2)

2 $$\frac{{Spee{d_{observed}}}}{{Spee{d_G}}}=1.06 \pm 0.36.$$

Combining nominal values predicted with Eq. (1) and standard deviations from Eq. (2), we obtain a representative range for the maximum speed expected from the GDS regression,

3 $$Spee{d_G}({\text{representative range}})={a_r}spee{d_{G({\mathrm{nominal}})}},$$

where *a*_*r*_ takes values between 0.70 and 1.42.

The model derived by Hirt et al.^20^ was based on body acceleration, muscle force and energy availability considerations, and it is written for land-moving animals as

4 $$Spee{d_H}({\mathrm{km/h}})=25.5{\left( {1000M} \right)^{0.26}}\left[ {1 - \exp \left( { - 22{{(1000M)}^{0.6}}} \right)} \right],$$

with the body mass is given in Tn. These authors quoted uncertainties in the coefficients (see their supplementary material) which give a significant amount of uncertainty for the maximum speed obtained (for example, the ratio between upper and lower bound results is close to a factor 4 and 6 for, respectively, *M* = 1 and 10 Tn), much higher than claimed by them. We evaluate the predictions from Eq. (4) in the same way as done with the G-regression, calculating the mean and standard deviation of the ratio between the observed and predicted speed for all the same 150 mammals in the database, obtaining (see Supplementary Table 3)

5 $$\frac{{Spee{d_{observed}}}}{{Spee{d_H}}}=1.12 \pm 0.38.$$

Combining nominal values predicted from Eq. (4) and standard deviations from Eq. (5), we obtain a representative range for the maximum speed expected from the HJRB model,

6 $$Spee{d_H}({\text{representative range}})={b_r}spee{d_{H({\mathrm{nominal}})}},$$

where *b*_*r*_ takes values between 0.74 and 1.50. We have performed the calculations for Eqs. (4), (5) and (6) using the database for land mammals (Supplementary Table 1) because the body mass-speed database of Hirt et al.^20^ includes data coming from many kinds of animals (e.g., insects) and several non-reliable sources^1^, including non-academic web pages.

### Accuracy of body mass-maximum speed relationships

The accuracy of both methods for estimating maximum speeds, the G-regression and the H-model, is very similar for mammals. The ratio between upper and lower bound results defined by Eqs. (3) and (6) have similar relative amplitudes of around a factor of 2. This relative amplitude range is clearly narrower than that calculated from parameter uncertainties quoted by Hirt et al.^20^ for Eq. (4) (see their Supplementary Table 3), and clearly wider than stated by these authors for quadrupedal dinosaurs from the 95% confidence interval of their model (which are similar to the relative amplitude of our representative ranges).

**Fig. 1:**
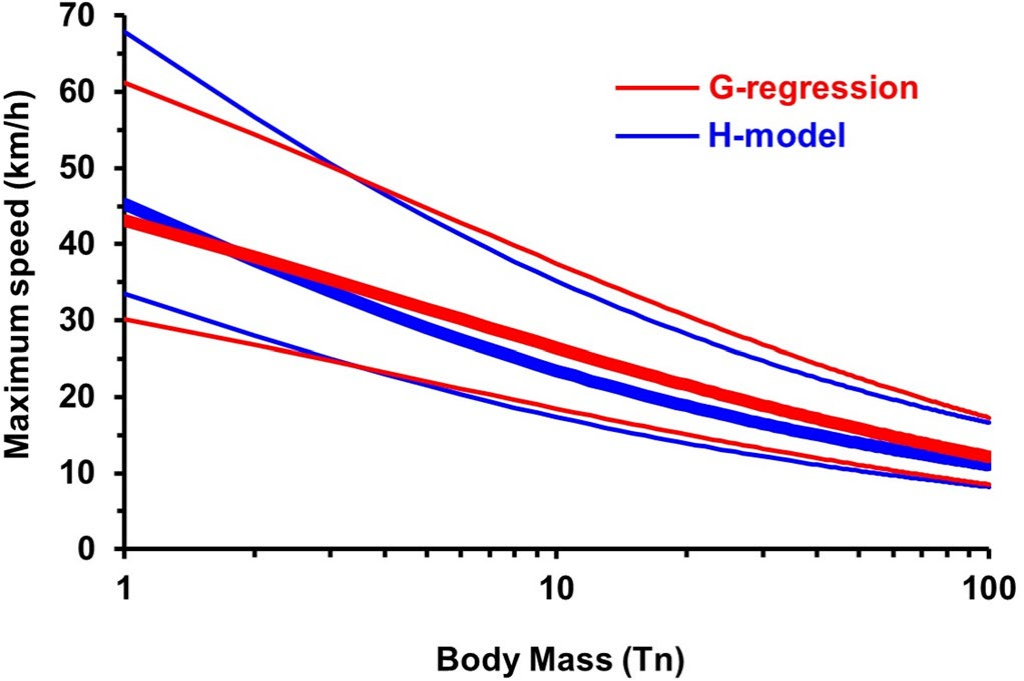
Maximum speed as a function of body mass calculated from both the G-regression and the H-model; see the main text for details. Thick curves indicate the prediction from both methods (given by Eqs. 1 and 4 respectively), whereas thin curves indicate the upper and lower limits when standard deviations are taken into account (given by Eqs. 3 and 6 respectively; see text for details).

Figure 1 compares the results obtained from Eqs. (1) and (4), as well as the respective upper and lower bounds obtained from Eqs. (3) and (6), for the mass range between 1 and 100 Tn. The differences between both methods are very similar for the extreme values in the range considered, and the greatest differences are for the interval between ~ 10 and ~ 30 Tn, but in no case the difference exceeds 3 km/h. A paired t-test comparing maximum speed predictions from the G-regression and the H-model for 20 large-bodied taxa (proboscideans and sauropods) revealed that speeds predicted by the G-regression were significantly higher by an average of 1.05 km/h (t = 22.93, df = 19, *p* < 0.0001). The 95% confidence interval for the mean difference was 0.95–1.14 km/h, and the effect size was substantial (Cohen’s d = 5.13).

It is important to recall that the maximum speed measured for a given animal does not discard that this animal might potentially be capable of reaching a faster speed. However, because moving on its potential highest speed is unusual for any animal, Eq. (1) to (6) are representative of the observed behaviors, and therefore we can use them for calculating probable maximum speeds for extinct animals.

### Extant proboscideans

**Fig. 2:**
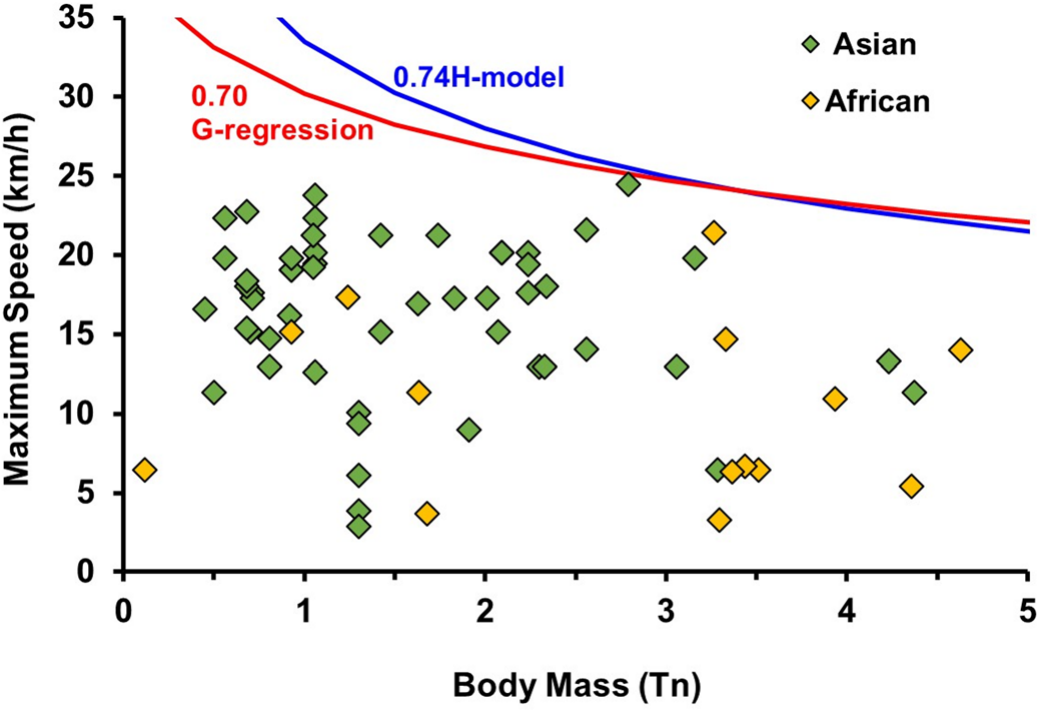
Maximum speed measured in controlled conditions for each one of the 68 elephants composing the database of Hutchinson et al.^25^. Also shown are the lower bounds of the predictions from both the G-regression and the H-model given, respectively, by Eq. (3) with *a*_*r*_ = 0.70, and Eq. (6) with *b*_*r*_ = 0.74 (see text for details).

Figure 2 shows that all maximum speeds measured for 68 live elephants (see Materials and Methods), 54 Asian elephants (*Elephas maximus*) and 14 African savannah elephants (*Loxodonta africana*), are clearly below the lower bounds of the ranges calculated from both the G-regression and the H-model. The kinematic differences between Asian and African elephants are very small^25^. Thus, we assume that the lower bound calculated from Eqs. (3) and (6) give reasonable upper limits to the maximum speed of large proboscideans.

Because it is unlikely that other large extinct graviportal animals were proportionally faster than proboscideans, we assume that those equations can be used to obtain upper bound estimates of the athletic capability of sauropods and ankylosaurs. Although the full range of results obtained with Eqs. (3) and (6) could be used for many animals, here we have found that the solutions for, respectively, *a*_*r*_ = 0.70 and *b*_*r*_ = 0.74 constitute empirical upper limits for live graviportal animals. However, this could not be the case for non-graviportal animals, and therefore we limit our application for extinct animals to graviportal ones.

### Maximum speeds for large extinct proboscideans

Table 1 shows maximum speeds for some selected large fossil proboscideans, calculated using both Eqs. (3) and (6), and taking, respectively, *a*_*r*_ = 0.70 and *b*_*r*_ = 0.74. Although there is a significant individual body size variability within species, we take average body mass estimates of Larramendi^26^, as representative for large-sized individuals of for each species. Given the degree of maximum speed variability in live elephants of similar body mass observed along the entire masses range shown in Fig. 2, and that all measured speeds are lower than predicted from Eqs. (3) and (6), the maximum speed values shown in Table 1 can be reliably considered as appropriate. *Mammuthus primigenius* (6 Tn) yielded estimated maximum speeds of 20.3 km/h using the H-model and 21.1 km/h from the G-regression, while the speed for larger *Mammut borsoni* (16 Tn) was calculated at 14.9 km/h and 16.1 km/h, respectively. Although the calculated maximum speeds for the larger proboscideans are comparatively lower, there is substantial overlap with those observed for the lighter extant proboscideans represented in Fig. 2. *Mammuthus* and *Palaeoloxodon* are phylogenetically closer to, respectively, Asian and African elephants^27^, and therefore we results are reliable for them (see ref. 25). For genera less closely related to extant proboscideans, such as *Mammut* or *Deinotherium*, our results are less reliable, although we consider them to be an interesting assessment of the athletic capability of extinct proboscideans.

**Table 1:** Maximum speed proposed for several giant fossil proboscideans, calculated from the lower bound of the ranges deduced in this work for the H-model, and for the G-regression. See main text for details.

	Representative mass (Tn) ^a^	Maximum speed (km/h) (H-model-based) ^b^	Maximum speed (km/h) (G-regression-based) ^c^
*Mammuthus primigenius*	6	20.3	21.1
*Mammut americanum*	8	18.6	19.6
*Mammuthus columbi*	9.5	17.6	18.7
*Deinotherium proavum*	10.5	17.1	18.2
*Mammuthus trogontherii*	11	16.8	18.0
*Mammuthus meridionalis*	11	16.8	18.0
*Palaeoloxodon antiquus*	13	16.0	17.2
*Mammut borsoni*	16	14.9	16.1

^a^ Average values for each species from ref. 26.

^b^ Maximum speeds calculated using Eq. (6) and *b*_*r*_ = 0.74.

^c^ Maximum speeds calculated using Eq. (3) and *a*_*r*_ = 0.70.

### Maximum speeds for giant sauropods

Sauropods are graviportal animals with columnar limbs (straight, pillar-like legs optimized for weight support), and some taxa are estimated to have body masses exceeding 50 Tn. Hirt et al.^20^ calculated, using their model, maximum speeds for sauropods of 28 and 78 Tn of, respectively 16.8 (9.8–21.1) and 12.0 (6.4–16.0) km/h; although these body masses were assigned by these authors to *Apatosaurus* and *Brachiosaurus*, respectively, the later value seems excessive^28,29^, but this point is beyond the scope of the present work.

**Fig. 3:**
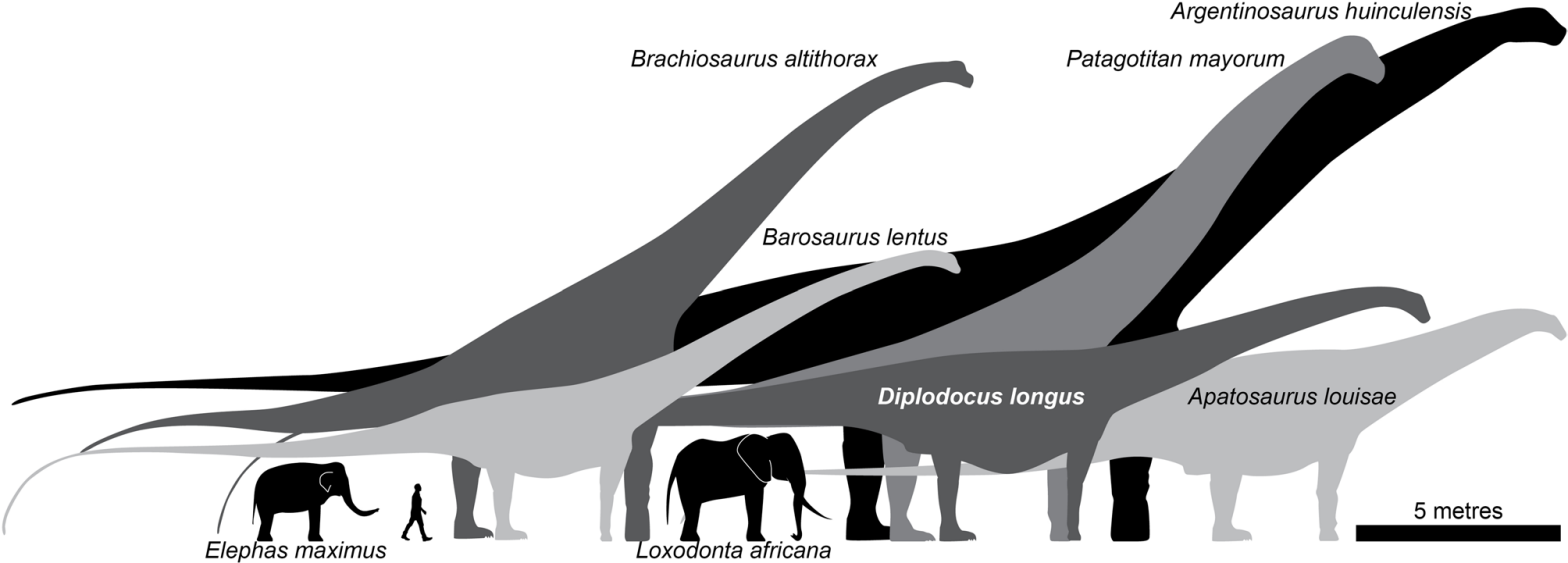
Several taxa of large sauropods whose athletic capabilities were analyzed in the present work, compared with Asian (*Elephas maximus*) and African savannah (*Loxodonta africana*) elephants, and a modern human (*Homo sapiens*). Note the large difference in body size between extant proboscideans and large sauropods.

Based on the analogy with the case of proboscideans, we propose to use the lower bounds for Eqs. (3) and (6) for calculate upper limits for the athletic capability of large sauropods, the archetypical graviportal extinct animals. We recognized that this proposal carries a large amount of extrapolation, because sauropods and proboscideans are separated by a large phylogenetic distance. Also, the larger sauropods can be an order of magnitude heavier than the elephants in the used database (Fig. 3). However, animals heavier than 0.5 Tn are consistently below the nominal predictions of the Eqs. (3) and (6), and for this reason we consider this extrapolation to be reasonable as a first-aproximation.

Table 2 shows maximum speed calculated in this way for several large (and popular) sauropods covering body masses^29,30^ between 10 and 75 Tn (i.e., *Dicraeosaurus* and *Argentinosaurus*, respectively). Macronaria sauropods include most of the largest ones (the examples in Table 2 are: *Camarasaurus*, *Turiasaurus*, *Brachiosaurus*, *Patagotitan* and *Argentinosaurus*), and they have forelimbs similar or larger than hindlimbs^31^; therefore, our calculations are less reliable than those for Diplodocoidea or basal sauropods, which usually have a more generalized locomotor body plan with hindlimbs being the largest. The obtained maximum speeds are modest: comparatively light sauropods (i.e., < 25 Tn, with speeds of 14–18.5 km/h) could might have reached speeds similar to some of the largest extinct proboscideans (e.g., *Mammut borsoni*, estimated at 16 Tn and 15–16 km/h). In contrast, gigantic taxa such as *Brachiosaurus* and *Patagotitan* could have obtained speeds of 10–11 km/h, while the largest taxon of our study (*Argentinosaurus*, estimated at 75 Tn) likely had maximum speeds below 10 km/h.

**Table 2:** Maximum speed proposed for several representative (and popular) sauropods, calculated following the same procedures as in Table 1. See main text for details.

	Representative mass (Tn) ^a^	Maximum speed (km/h) (H-model-based) ^b^	Maximum speed (km/h) (G-regression-based) ^c^
*Dicraeosaurus sattleri*	10	17.4	18.5
*Barosaurus lentus*	13	16.0	17.2
*Diplodocus longus*	15	15.3	16.5
*Camarasaurus grandis*	18	14.4	15.6
*Antarctosaurus brasilensis*	20	13.9	15.1
*Cetiosaurus oxoniensis*	25	12.9	14.0
*Apatosaurus louisae*	34	11.7	12.7
*Turiasaurus riodevensis*	42	10.9	11.8
*Brachiosaurus altithorax*	47	10.5	11.3
*Patagotitan mayorum*	56	9.9	10.6
*Argentinosaurus hiunculensis*	75	9.0	9.5

^a^ Body masses below 30 Tn from ref. 29; body masses above 30 Tn taken as the preferred values from ref. 28.

^b^ Maximum speeds calculated using Eq. (6) and *b*_*r*_ = 0.74.

^c^ Maximum speeds calculated using Eq. (3) and *a*_*r*_ = 0.70.

Some researchers suggest that the body masses of sauropods, including those examined in the current study, may have been significantly higher than the estimates used here. For example, a body mass up to 95 Tn has been proposed^32^ for *Argentinosaurus* (compared to the here used estimate of 75 Tn). If these sauropods were indeed heavier, it is likely that their maximum speeds would have been even lower than our current estimates (less than 9 km/h for a body mass of 95 Tn).

### The case of large ankylosaurs

Large ankylosaurs have been also considered as graviportal animals^33,34^, although in this case the comparatively low body (specifically, the low hip height relative to body mass^33,35,36^) suggests that the athletic capabilities of ankylosaurs could have been lower than those of live elephants of similar body mass. If we accept ankylosaurs as graviportal animals, and we use the lower bounds of Eqs. (3) and (6) for an *Ankylosaurus magniventris* of 4.8 Tn (ref. 29), a maximum speed of around 22 km/h would be obtained, which seems unlikely considering the robust morphology and comparatively short limbs of this animal.

Ankylosaurids have a distinctive body plan that included, not only an appendicular musculoskeletal system that allowed them to acquire a quadrupedal stance and graviportal locomotion, but a considerable and complex dermal armor that could have influenced their locomotion^36^. This complex ornamentation that covered a large part of the body of these dinosaurs could have added additional mass and change the location of the center of mass^37^. One difference of ankylosaurs compared with mammals or sauropods is that quadrupedal ornithischian forelimbs are quite notably shorter than their hindlimbs^37^. Furthermore, ornithischian dinosaurs had other features in their forelimbs such bent elbows, that could have limited the protraction of their humerus beyond vertical, as well as a rigid scapula that would not have contribute significantly during the locomotion^34,37^. Taking all of this into account it is reasonable to think that the maximum speed of ankylosaurs would have been lower than predicted by Eqs. (3) and (6).

## Discussion

The relation between body mass and maximum speed may be used to constrain the athletic capability of large graviportal animals. It is worth making clear that both the G-regression and the H-model were derived from datasets encompassing species with different locomotor adaptations. However, we constrain here their applications with data from live graviportal animals, and therefore our results are only valid for graviportal animals. For non-graviportal land animals we could use the Eqs. (3) and (6) taking the whole range of possible values for, respectively, *a*_*r*_ and *b*_*r*_.

In this work we have calculated upper limits for the maximum speeds, related to body mass, of diverse taxa of large graviportal animals, not the actual speeds for those animals. Thus, our calculations search for upper limits to maximum speed, and by definition, an upper limit does not have any uncertainty range or error bars associated. The actual maximum speed for a given large graviportal animal would be equal or lower than our calculated value. Limitations imposed by body plan, limb morphology or metabolism will impose other restrictions to the speed of animals^38^, but those restrictions are beyond the scope of the present work. The speed calculated here must be therefore taken as approximate values, which should not be seen as an attempt to state that a given taxon would have an exact athletic capability, or that two taxa with the same body mass would reach similar speeds. Thus, our results do not pretend to be a definitive conclusion on the athletic capability of large proboscideans or sauropods: We simply stated that body mass-based considerations suggest that the analyzed animals most probably did not surpass the calculated speed.

The 68 live elephants composing the database of Hutchinson et al.^25^ always moved below the representative range expected from both the H-model and the G-regression, despite targeting highest locomotor performance for each individual. As above indicated, we consider these equations appropriate for calculating maximum speed of proboscideans, moreover taking into account that much of the faster Asian elephants in the data base of Hutchinson et al.^25^ were Thai elephants trained for work and activities requiring speed. In any case, the scatter of body mass-maximum speed points for those animals (see Fig. 2) is very wide, although it might partly be due to individual behavior, and not only to individual variability in athletic capability. Similar variability has been reported in other large mammals such as (cursorial) giraffes^39^.

It has been argued that larger elephants avoid maximal locomotory intensity (such as applying maximal stride length) in order to avoid excessive bending and torsion of bones^25,40^. This is likely the reason for the average lower maximum speed in large proboscideans compared to smaller ones, despite the larger species having longer limbs and therefore longer maximum stride lengths in theory. Smaller elephants are more agile, and they are able to compensate for smaller absolute stride lengths by adopting increased stride cadences and applying maximal stride lengths for their size, in comparison to larger elephants^25^.

When extrapolating both the H-model and the G-regression to extinct proboscideans of body mass larger than present-day ones, the predicted maximum speeds lie within the range of maximum speeds measured for Asian and African elephants. This indicates that proboscideans spanning a wide range of body masses may have had comparable maximum speeds, and that individual variability is probably high, due to both behavior and actual athletic capability.

While our extrapolations regarding maximum speeds in sauropods carry some uncertainties, the use of the lower bounds of Eqs. (3) and (6) for these large, graviportal animals can be useful to provide upper limit estimates of their locomotor capabilities. Although the G-regression consistently predicts maximum speeds approximately 1 km/h higher than the H-model, both models, when bounded by empirical data from live proboscideans, yield consistent conclusions regarding the limited athletic capabilities of giant fossil taxa. For the largest sauropods, particularly those approaching or exceeding 50 Tn, our calculated maximum speeds are around 10 km/h, with all evaluated taxa (spanning 10–75 Tn) having estimated maximum speeds below 20 km/h. These findings suggest that the immense body size and graviportal body built of sauropods were key factors that likely restricted their locomotion to a single, steady gait (i.e., walking), unlike smaller animals—and even their ancestral forms—which could transition to faster gaits such as trotting or running. Rather than relying on speed, sauropods may have evolved to sustain energy-efficient walking^41,42^, which likely allowed them to cover large areas while foraging for food and water. Their massive size was an asset, enabling them to dominate within their Mesozoic ecosystems. Trackway evidence supports this interpretation, as trackways attributed to titanosaurs indicate walking speeds below 5 km/h (e.g., ref^43,44^.), further supporting that these massive animals prioritized slower-paced movement.

The results presented in this work are interesting as a first assessment of the athletic capabilities of very large animals. Indeed, the relationship between body mass and maximum speed is well established, but the comparison of the predictions from both the G-regression and H-model with observational data from live elephants indicates that the athletic capabilities of giant graviportal extinct animals were more probably below the lower bound of the standard deviation of those predictions. Moreover, the individual variability observed in live elephants suggests that the actual athletic capability of many individuals of extinct species in Tables 1 and 2 may have been lower than the calculations presented in this work. Finally, because our analysis is only addressed for animals usually recognized as graviportal, we must be cautious to extrapolate our results to other giant fossil quadruped animals, such as the largest rhinoceroses or ceratopsians.

## Materials and methods

### Mass-maximum speed relationship in live proboscideans

We compare our results with a database of speeds of live proboscideans measured in controlled settings^25,45^. This database contains 54 Asian elephants (*Elephas maximus*) and 14 African savannah elephants (*Loxodonta africana*). We only use the highest speed recorded for each given animal (see Supplementary Table 4), because we are dealing with theoretical maximum speeds. Body masses expand the range between 0.1 and 4.6 Tn, and maximum speeds of up to 24.5 km/h. Maximum speeds for Asian and African elephants are roughly similar (although the later ones are somewhat slower for a same mass).

Hutchinson et al.^25^ demonstrated a strong similarity between the kinematics of Asian and African elephants, and therefore these authors consider these animals of utility for studying the athletic capability of extinct proboscideans. Much of the Asian Elephant were “working” Thai elephants trained by fast activities, including elephant races, chasing other elephants, and polo matches. For this reason, the very high speeds reported in times for some free elephants (e.g., 40 km/h) were considered by Hutchinson et al.^25^ as unrepresentative, and probably dues to the difficulty of estimating speed and animal size in non-controlled settings.

### Large fossil proboscideans

For extinct proboscideans, maximum speeds were calculated by applying the lower bounds of both the H-model (Eq. 6; *b*_*r*_ = 0.74) and the G-regression (Eq. 3; *a*_*r*_ = 0.70). Representative body masses for each species were obtained from Larramendi^26^, covering a range from 6 to 16 tonnes. The species included in this study were *Mammuthus primigenius* (6 Tn), *Mammut americanum* (8 Tn), *Mammuthus columbi* (9.5 Tn), *Deinotherium proavum* (10.5 Tn), *Mammuthus trogontherii* (11 Tn), *Mammuthus meridionalis* (11 Tn), *Palaeoloxodon antiquus* (13 Tn), and *Mammut borsoni* (16 Tn). Larramendi^26^ used a volumetric method, and concentrated on large-sized individuals of advanced age, and therefore most of their body mass values (except perhaps for *M. columbi*, *D. proavum*, and *M. trogontherii*), are likely closer to maximum rather than average body masses. Because we are interested in body masses considered representative for the respective species, it is not necessary to search for a large precision in the mass estimates (obviously a species includes members of different body mass).

### Giant sauropods

Maximum speeds for sauropod dinosaurs were estimated using the same procedure, applying the lower bounds of the H-model (Eq. 6; *b*_*r*_ = 0.74) and the G-regression (Eq. 3; *a*_*r*_ = 0.70). Representative body masses were sourced from Benson et al.^30^ for taxa with masses below 30 tonnes, and from Campione and Evans^29^ for those above this threshold, spanning a range from 10 to 75 tons. We select sauropods with a wide range of body mass, including very heavy examples. Although some species have more complete skeletal records than other ones, we select values from two papers calculating body masses based on allometric relations. A recent paper^46^ using a volumetric approximation obtained body masses for some sauropods in Table 2 in general highest than the values here used, although with uncertainty ranges including them. Again, we are more interested in body masses considered as representative for different species than in exact estimates for specific individual of those species. The analysed sauropod taxa included *Dicraeosaurus sattleri* (10 Tn), *Barosaurus lentus* (13 Tn), *Diplodocus longus* (15 Tn), *Camarasaurus grandis* (18 Tn), *Antarctosaurus brasilensis* (20 Tn), *Cetiosaurus oxoniensis* (25 Tn), *Apatosaurus louisae* (34 Tn), *Turiasaurus riodevensis* (42 Tn), *Brachiosaurus altithorax* (47 Tn), *Patagotitan mayorum* (56 Tn), and *Argentinosaurus huinculensis* (75 Tn). These species were selected to represent a range of sauropod body sizes and clades.

## Supplementary Information

Below is the link to the electronic supplementary material.

Supplementary Material 1

Supplementary Material 2

Supplementary Material 3

Supplementary Material 4

## Data Availability

The data supporting this study are all presented in Supplementary Tables 1 to 4.
